# Antenatal depression and anxiety and early pregnancy BMI among White British and South Asian women: retrospective analysis of data from the Born in Bradford cohort

**DOI:** 10.1186/s12884-020-03097-2

**Published:** 2020-09-01

**Authors:** Nafisa Insan, Emma Slack, Nicola Heslehurst, Judith Rankin

**Affiliations:** Population Health Sciences Institute, Newcastle University, Newcastle upon Tyne, NE2 4AX UK

**Keywords:** Body mass index, Depression, Anxiety, Obesity, Pregnancy, White British, South Asian

## Abstract

**Background:**

Maternal obesity has severe physical impacts such as increased chances of pre-eclampsia and gestational diabetes. However, mental health impacts are given less attention within antenatal care. Evidence suggests that women with obesity carry increased risk of maternal depression and anxiety, however, this association is not well researched amongst South Asian women in the UK who are vulnerable to both. The aim of this study was to investigate the association between antenatal depression and anxiety and early pregnancy BMI, within and between White British and South Asian women, using data from the Born in Bradford cohort.

**Methods:**

Depression and anxiety were assessed using the General Health Questionnaire (GHQ); a GHQ score of > 0 for the depression subscale and > 6 for anxiety. Mother’s BMI was stratified into six World Health Organisation BMI categories (underweight, recommended, overweight or obese class 1–3). To determine associations, univariate and multivariate logistic regression models (adjusting for maternal age, education, deprivation and smoking) were used.

**Results:**

There were 7824 women included (3514 White British and 4310 South Asian). South Asian women were more likely to have depression than White British (43.3% vs 36.1% *p* < 0.0001) and less likely to have anxiety (45.3% vs 48.4% *p* < 0.01). There were no significant associations between BMI and depression or anxiety in South Asian women. White British women with an overweight BMI had higher odds of anxiety compared with women with a recommended BMI (Adjusted Odds Ratio 1.25, 95% Confidence Interval 1.05–1.47). No significant associations were observed for other BMI categories. Smoking was a risk factor for antenatal depression (AOR 1.32, 95% CI 1.12–1.56; AOR 2.08, 95% CI 1.49–2.91) and anxiety (AOR 1.34, 95% CI 1.14–1.57; (AOR 2.87, 95% CI 2.02–4.07) in both White British and South Asian women, respectively.

**Conclusions:**

Although South Asian women have a higher prevalence of depression than White women in this cohort, the known associations between maternal obesity and anxiety do not appear to be present. More studies are needed using validated depression tools for South Asian pregnant women. Mental health screening during antenatal care is important for South Asian women, with factors such as smoking considered.

## Background

Depression and anxiety are the most common mental health conditions during pregnancy, with approximately 12 and 13% of women experiencing them, respectively [1]. Antenatal depression and anxiety are associated with increased risk of post-partum depression, and poor cognitive and emotional development in the child [2]. South Asian (Indian, Pakistani and Bangladeshi) women are more susceptible to depression and anxiety compared to White British women [3].

There is evidence of a significant association between mental health conditions during pregnancy and maternal obesity. A systematic review and meta-analysis published in 2014 found odds of antenatal depression and anxiety increased by 43 and 30%, respectively, in women with obesity compared to women of recommended BMI [4]. Two studies conducted in 2017 [5] and 2018 [6] in Finland also found significantly increased odds of anenatal depression by 51 and 43% respectively, in women with early pregnancy (12-13 weeks gestation) obesity. However, a 2017 study [7]  in the United States found no significant association between obesity and antenatal depression. These previous studies were predominately from high-income countries (HICs), with 53 out of 62 studies in the 2014 systematic review [4]. Further, only two studies included in the systematic review [4] focused on ethnic-minorities (African American and Hispanic) [8, 9]; while none focused on South Asian women. A systematic review [10] investigating the effects of maternal anthropometrics on pregnancy outcomes in South Asian women did not identify any studies on mental health conditions, further highlighting the lack of published data in this field in South Asian women.

In addition to the increased risk of mental health conditions among South Asian women, this population also carry an increased risk of maternal obesity. In the UK, half of all pregnant women enter pregnancy with a BMI in the overweight or obese range [11]. Evidence suggests that South Asian women have the highest odds of first trimester obesity compared to White British women [12], and an increased risk of obesity-related complications, e.g. Gestational Diabetes Mellitus [7] during pregnancy at a lower BMI than White British women. The World Health Organisation (WHO) reflects this in their BMI criteria, where the categories for overweight and obesity are lower in South Asian groups (23–27.49 kg/m^2^ and ≥ 27.5 kg/m^2^ respectively) [10] compared with the general population criteria (25–29.9 kg/m^2^ and ≥ 30 kg/m^2^) [13]. The National Institute of Health and Care Excellence guidelines have recommended the use of these lower WHO thresholds to identify and treat South Asian individuals with obesity-related illnesses [14]; however, this is not reflected in pregnancy guidelines.

Given the increased risk for both obesity and mental health conditions in South Asian women, compared to White British, and the limited research focusing on these ethnic differences, the aim of this study was to address the current research gap by investigating the association between antenatal depression and anxiety and early pregnancy BMI within and between South Asian and White British women, using data from the Born in Bradford (BiB) cohort. We hypothesised that higher early pregnancy BMI, particularly obesity, would be associated with antenatal depression and anxiety, with South Asian women displaying higher risk than White British women.

## Methods

### Study population

Bradford is the seventh largest city in the UK with a population of 537200 in 2018 and a growth rate of 0.06% [15]. This growth is largely due to the higher number of births than deaths; in 2017 there were 3600 more births than deaths. Bradford is one of the most deproved areas in the UK [15]. Around 20% of the popualtion are of South Asian origin, with higher fertility rates compared with the White British population. Almost half of the babies born in the city have parents of Pakistani origin and form a significant proportion of the individuals with poor health outcomes in the city [15]. BiB is a longitudinal multi-ethnic community birth cohort of 12453 women (including 6900 South Asian, 50.1%) established in Bradford in 2007 to understand the reasons behind poor health outcomes within the city [15]. The cohort aimed to examine how environmental, genetic, behavioural and social factors impact on the health of the mother and child, and development from childhood through to adult life [15]. Many research studies using the BiB cohort put particular emphasis on investigating differences between White British and South Asian individuals to explore important health inequalities, and to provide culturally appropriate healthcare within this population [15].

### Study design

This study was a retrospective analysis of data from the BiB cohort. White British and South Asian pregnant women were the populations of interest. Women from other ethnic groups, or where ethnicity data were missing, were excluded from the analysis. The BiB questionnaire was administered to women in a standardised manner during 26-28 weeks gestation when they attended for an oral glucose tolerance test [15]. The original questionnaire was translated into Urdu and Mirpuri for women of South Asian origin who could not read or speak English and this was verbally administered [15]. This was carried out in a standardised process to ensure accurate translation using bilingual translators [15]. No incentives were offered for participation reducing self-reporting bias [15].

The dependent variables were the General Health Questionnaire (GHQ) scores indicating antenatal depression and anxiety. The GHQ is a validated self-report questionnaire with 28 items relating to the respondent’s current mental state, ability to carry out functions and daily activities and appearance of new and distressing phenomena [16]. The 28 items are grouped into four categories each used to identify symptoms of certain psychiatric disorders. Since this study focused on depression and anxiety as the most common mental health conditions during pregnancy, the total Likert score of the subscales for depression and anxiety (D, items 22–28; B, items 8–14) were used in the analysis (see Additional File 1) [16]. There are no agreed thresholds for the subscales to indicate depression or anxiety, but studies suggest that the cut-off score should be based on the mean/median of the sample of interest [17, 18]. In this study, the median was used due to the non-normality of distribution. This was 0 for depression and 6 for anxiety. Therefore, a score of > 0 was used to indicate depression and > 6 indicated anxiety.

The main independent variable was maternal early pregnancy BMI. Data from the BiB cohort includes information on the mother’s booking BMI calculated using measured height and weight between 10 and 12 weeks of pregnancy [19]. A realistic lower limit of 11 kg/m^2^ was set as this has been shown to be the lowest BMI for survival in women [20]. An upper limit of 80 kg/m^2^ was based on the frequency distribution in the data from the BiB cohort and a published study [21]. Women with a booking BMI outside this range were excluded from analysis (*n* = 720, 6.5%). BMI was analysed as a categorical variable due to the inclusion of underweight which is also associated with increased depression and anxiety [22], therefore, a continuous analysis may skew the results. BMI was stratified by the WHO’s classification. For White British women, the categories were: underweight, < 18.5 kg/m^2^; recommended weight, 18.5–24.9 kg/m^2^; overweight, 25–29.9 kg/m^2^; and obese class 1, 30.0–34.9 kg/m^2^; class 2, 35.0–39.9 kg/m^2^; class 3, ≥40.0 kg/m^2^ [2]. The categories used for South Asian women were: underweight, < 18.5 kg/m^2^; recommended weight, 18.5–22.9 kg/m^2^; overweight, 23–27.49 kg/m^2^; and obese class 1, 27.5–32.49 kg/m^2^; class 2, 32.5–37.49 kg/m^2^; class 3, ≥37.5 kg/m^2^ [23]. A secondary analysis was performed using the general population BMI criteria (see Additional File 2) for South Asian women due to the current lack of guidance in the UK for using Asian-specific criteria in pregnancy.

Additional variables included in the adjusted models were maternal age, maternal education, area of residence deprivation (based on postcode) and maternal smoking. Maternal age (years) was analysed as a continuous variable. Maternal education was defined as mother’s highest educational qualification (equivalised) with the following categories: none, GSCE equivalent, A-level equivalent and higher than A-level (used as reference group). The Index of Multiple Deprivation (IMD) was used to categorise area of residence deprivation. The IMD is a measure of relative deprivation for small areas in England and is the most widely used tool to measure deprivation in health-related research in the UK [24]. Since BiB was carried out in Bradford, the national IMD quintiles would be of limited use for this study because Bradford has a higher level of deprivation compared to most areas of the UK [15]. Therefore, the deprivation data were skewed towards the most deprived quintiles for this population. A binary variable was created with quintiles 2–5 combined to represent lower levels of deprivation (used as the reference group) and quintile 1 represented the highest level of deprivation. Maternal smoking during pregnancy was a binary variable (yes/no). Full details for all variables can be found online in the BiB Data Dictionary [25].

### Statistical analysis

Primarily, data were summarised using descriptive statistics (proportions and percentages for categorical variables and means and standard deviations for continious variables). Following this, univariate independent t-tests were used to test for significant associations for normally distributed continuous variables and the chi-squared and Fishers exact test were used for categorical variables. Statistical hypotheses were tested using two-tailed 95% confidence intervals (95% CI), with a signficance level of 0.05 and critical value of 1.96 set. Regression assumptions were met, and data were analysed using multiple logistic regression modelling with maternal BMI, maternal age, maternal education, deprivation level and smoking during pregnancy as independent variables, and GHQ depression > 0 and GHQ anxiety > 6 as the dichotomous dependent variables. Finally, interaction analysis was carried out to determine if there were differences in the regression associations between White British and South Asian women i.e. associations between ethnicity and antenatal depression and anxiety within each BMI category. The analyses were performed using STATA version 16.

## Results

There were 9420 White British and South Asian women who had a BMI between 11 and 80 kg/m^2^. Following exclusions for missing GHQ data (*n* = 1596, 17%), a total sample size of 7824 women was available for analysis. One reason for this missing data is during the first few months of recruitment, the GHQ was not included in the questionnaire. Also, some women may have chosen not to answer some of the questions. The final sample included 3514 White British and 4310 South Asian women (3780 Pakistani, 343 Indian and 187 Bangladeshi). There was no statistically significant difference between the included and excluded group in terms of age (27.3 ± 0.1 versus 27.3 ± 0.14) or deprivation level (low deprivation 34.5% vs 31.3%, high deprivation 65.5% vs 68.7%) (Table 1). However, the included population had significantly higher education levels than those excluded (>A-level 24.6% vs 21.1%, A-level 15.8% vs 8.9%, *p* < 0.0001) and were more likely to smoke (17.6% vs 10.2%, p < 0.0001) (Table 1).

**Table 1:** Difference in maternal characteristics in the included and excluded groups

Maternal characteristics	Included *n* = 7824	Excluded *n* = 1596	Differences in mean or %	*p*-value^p^
Age (mean, SD)	27.3 ± 0.1	27.3 ± 0.14	0	0.96
Education level (n, %)				
None	1685 (21.5)	424 (26.6)	+ 5.1	< 0.0001**
GCSE	2475 (31.6)	535 (33.5)	+ 1.9	
A-level	1235 (15.8)	142 (8.9)	−6.9	
Higher than A-level	1924 (24.6)	337 (21.1)	−3.5	
*Missing*	*505 (6.4)*	158 (9.9)	+ 3.5	
IMD Level (n, %)				
Low deprivation	2696 (34.5)	500 (31.3)	−3.2	0.16
High deprivation	5125 (65.5)	1096 (68.7)	+ 3.2	
*Missing*	*3 (0.04)*	0 (0.0)	−0.04	
Smoking status (n, %)				
Yes	1375 (17.6)	162 (10.2)	−7.4	< 0.0001**
No	6446 (82.4)	1427 (89.4)	+ 7.0	
*Missing*	*3 (0.04)*	7 (0.4)	+ 0.36	

**Significant at 0.05 significance level (two-tailed)

^p^T-tests used for continuous variable and chi-squared with Fishers exact (where cell count < 5) used for categorical variables

Table 2 shows the summary of characteristics of the included sample of pregnant women, stratified by ethnicity, with tests for association between the ethnic groups. In the included population, South Asian women were significantly older than White British women, had a higher percentage with >A-level education level, were more likely to live in the highest deprivation areas, less likely to smoke, had a higher proportion with depression, but a lower proportion with anxiety, independent of early pregnancy BMI. There was a significant association between BMI and ethnicity, with a higher proportion of South Asian than White British women in the overweight and obese categories (Table 2).

**Table 2:** Maternal characteristics of all women stratified by ethnicity

	Total *n* = 7824	White British *n* = 3514	South Asian *n* = 4310	*p*-value^p^
BMI categories^+^ (n, %)				
Underweight	336 (4.3)	94 (2.7)	242 (5.6)	< 0.0001**
Recommended	2835 (36.2)	1514 (43.1)	1321 (30.6)	
Overweight	2438 (31.2)	1003 (28.5)	1435 (33.3)	
Obese	2215 (28.3)	903 (25.7)	1312 (30.4)	
Class 1	1361 (17.4)	508 (14.5)	853 (19.8)	
Class 2	589 (7.5)	264 (7.5)	325 (7.5)	
Class 3	265 (3.4)	131 (3.7)	134 (3.1)	
Age (mean, SD)	Mean = 27.3 ± 0.1	26.6 ± 0.1	27.8 ± 0.1	< 0.0001**
Education level (n, %)				
None	1685 (21.5)	698 (19.9)	987 (22.9)	< 0.0001**
GCSE	2475 (31.6)	1192 (33.9)	1283 (29.8)	
A-level	1235 (15.8)	645 (18.3)	590 (13.7)	
Higher than A-Level	1924 (24.6)	678 (19.3)	1246 (28.9)	
*Missing*	*505 (6.4)*	*301 (8.6)*	*204 (4.7)*	
IMD level (n, %)				
Low deprivation	2696 (34.5)	1702 (48.4)	994 (23.1)	< 0.0001**
High deprivation	5125 (65.5)	1810 (51.5)	3315 (76.9)	
*Missing*	*3 (0.04)*	*2 (0.1)*	*1 (0.02)*	
Smoking status (n, %)				
Yes	1375 (17.6)	1217 (34.6)	158 (3.7)	< 0.0001**
No	6446 (82.4)	2295 (65.3)	4151 (96.3)	
*Missing*	*3 (0.04)*	*2 (0.1)*	*1 (0.02)*	
Depression (n, %)				
Yes	3158 (40.4)	1267 (36.1)	1891 (43.9)	< 0.0001**
No	4666 (59.6)	2247 (63.9)	2419 (56.1)	
Anxiety (n, %)				
Yes	3655 (46.7)	1701 (48.4)	1954 (45.3)	0.004**
No	4113 (52.6)	1790 (22.9)	2323 (53.9)	
*Missing*	*56 (0.7)*	*23 (0.3)*	*33 (0.8)*	

*SD* standard deviation

*GCSE* The General Certificate of Secondary Education

*IMD* Index of Multiple deprivation

^+^White British BMI categories: Underweight, < 18.5 kg/m^2^; recommended weight, 18.5–24.9 kg/m^2^; overweight, 25–29.9 kg/m^2^; obese, ≥30 kg/m^2^; obese class 1, 30.0–34.9 kg/m^2^; obese class 2, 35.0–39.9 kg/m^2^; obese class 3, ≥40.0 kg/m^2^ South Asian BMI categories: underweight, < 18.5 kg/m^2^; recommended weight, 18.5–22.9 kg/m^2^; overweight, 23–27.49 kg/m^2^; obese, ≥27.5 kg/m^2^; obese class 1, 27.5–32.49 kg/m^2^, obese class 2, 32.5–37.49 kg/m^2^, obese class 3, ≥37.5 kg/m^2^

**Significant at 0.01 significance level (two-tailed)

^p^T-test for continuous variable and chi-squared with Fishers exact (where cell count < 5) for categorical variables

Missing data not included in chi-squared analysis

Within the White British ethnic group, women with obesity were significantly older, had a lower percentage with >A-level education level, were more deprived and less likely to smoke, compared to women with a recommended BMI (Table 3). Overall, 36% of White British women had depression and 48% had anxiety. Within South Asian women, those with obesity were significantly older and had a lower percentage with >A-level education level compared with women with a recommended BMI. There was no significant association between deprivation level, smoking status, depression or anxiety and early pregnancy BMI among South Asian women. In both the South Asian and White British groups, women with an obese BMI had higher proportions with depression and anxiety, compared with women with a recommended BMI, although this was not statistically significant.

**Table 3:** Maternal characteristics of White British and South Asian women, stratified by BMI^+^

	Underweight		Recommended weight		Overweight		Obese class 1		Obese class 2		Obese class 3		*p-*value^p^	
	White British *n* = 94	South Asian *n* = 242	White British *n* = 1514	South Asian *n* = 1321	White British *n* = 1003	South Asian *n* = 1435	White British *n* = 508	South Asian *n* = 853	White British *n* = 264	South Asian *n* = 325	White British *n* = 131	South Asian *n* = 134	White British	South Asian
Age (mean ± SD)	21.8 ± 0.5	25.2 ± 0.3	25.7 ± 0.2	26.4 ± 0.1	27.4 ± 0.2	28.2 ± 0.1	27.7 ± 0.2	29.0 ± 0.2	27.6 ± 0.3	29.2 ± 0.3	28.2 ± 0.5	30.8 ± 0.5	0.0001**	0.0001**
Education level (n, %)														
None	28 (29.8)	50 (20.7)	329 (21.7)	255 (19.3)	169 (16.8)	321 (22.4)	88 (17.3)	212 (24.9)	55 (20.8)	100 (30.8)	29 (22.1)	49 (36.6)	< 0.0001	< 0.0001
GSCE	43 (45.7)	80 (33.1)	512 (33.8)	392 (29.7)	328 (32.7)	406 (28.3)	173 (34.1)	262 (30.7)	90 (34.1)	102 (31.4)	46 (35.1)	42 (31.3)	**	**
A-level	12 (12.8)	27 (11.2)	247 (16.3)	186 (14.1)	202 (20.1)	202 (14.1)	103 (20.3)	120 (14.1)	58 (22.0)	41 (12.6)	23 (17.6)	14 (10.4)		
Higher than A-Level	7 (7.4)	76 (31.4)	316 (20.9)	428 (32.4)	210 (20.9)	439 (30.6)	91 (17.9)	223 (26.1)	32 (12.1)	65 (20.0)	22 (16.8)	15 (11.2)		
IMD level (n, %)														
Low deprivation	24 (25.5)	55 (22.7)	765 (50.5)	330 (25.0)	535 (53.3)	349 (24.3)	234 (46.1)	179 (21.0)	95 (36.0)	60 (18.5)	49 (37.4)	21 (15.7)	< 0.0001**	0.015*
High deprivation	70 (74.5)	186 (76.9)	748 (49.4)	991 (75.0)	468 (46.7)	1086 (75.7)	273 (53.7)	674 (79.0)	169 (64.0)	265 (81.5)	82 (62.6)	113 (84.3)		
Smoking status (n, %)														
Yes	56 (59.6)	6 (2.5)	542 (35.8)	48 (3.6)	322 (32.1)	63 (4.4)	165 (32.5)	21 (2.5)	98 (37.1)	14 (4.3)	34 (26.0)	6 (4.5)	< 0.0001**	0.181
No	38 (40.4)	236 (97.5)	970 (64.1)	1273 (96.4)	681 67.9)	1371 (95.5)	343 (67.5)	832 (97.5)	166 (62.9)	311 (95.7)	97 (74.0)	128 (95.5)		
Depression (n, %)														
Yes	37 (39.4)	97 (40.1)	543 (35.9)	555 (42.0)	352 (35.1)	657 (45.8)	179 (35.2)	380 (44.5)	107 (40.5)	137 (42.2)	49 (37.4)	65 (48.5)	0.630	0.212
No	57 (60.6)	145 (59.9)	971 (64.1)	766 (58.0)	651 (64.9)	778 (54.2)	329 (64.8)	473 (55.5)	157 (59.5)	188 (57.8)	82 (62.6)	69 (51.5)		
Anxiety (n, %)														
Yes	38 (40.4)	95 (39.3)	699 (46.2)	581 (44.0)	510 (50.8)	657 (45.8)	250 (49.2)	405 (47.5)	131 (49.6)	153 (47.1)	73 (55.7)	63 (47.0)	0.065	0.261
No	55 (58.5)	146 (60.3)	803 (53.0)	726 (55.0)	487 (48.6)	764 (53.2)	257 (50.6)	446 (52.3)	130 (49.2)	171 (52.6)	58 (44.3)	70 (52.2)		

*SD* standard deviation

*GCSE* The General Certificate of Secondary Education

*IMD* Index of Multiple deprivation

*Significant at 0.05 significance level (two-tailed)

**significant at 0.01 significance level (two-tailed)

^p^T-test for continuous variable and chi-squared with Fishers exact (where cell count < 5) for categorical variables

^+^White British BMI categories: Underweight, < 18.5 kg/m^2^; recommended weight, 18.5–24.9 kg/m^2^; overweight, 25–29.9 kg/m^2^; obese, ≥30 kg/m^2^; obese class 1, 30.0–34.9 kg/m^2^; obese class 2, 35.0–39.9 kg/m^2^; obese class 3, ≥40.0 kg/m^2^ South Asian BMI categories: underweight, < 18.5 kg/m^2^; recommended weight, 18.5–22.9 kg/m^2^; overweight, 23–27.49 kg/m^2^; obese, ≥27.5 kg/m^2^; obese class 1, 27.5–32.49 kg/m^2^, obese class 2, 32.5–37.49 kg/m^2^, obese class 3, ≥37.5 kg/m^2^

Sum of percentages may not add to 100% due to missing/incomplete data, missing data not included in chi-squared analysis

There was no significant association between depression and early pregnancy BMI among White British women (Table 4). Univariate regression found that South Asian women with an overweight BMI had significantly higher odds of depression than South Asian women of recommended BMI (odds ratio (OR) 1.17, 95% CI 1.00–1.36). After adjusting for age, education level, smoking and deprivation, the adjusted odds ratio (AOR) was no longer significant (AOR 1.16, 95% CI 0.99–1.36). No other associations between BMI and depression were found to be significant. Interaction analysis found no significant association between ethnicity and antenatal depression within any BMI category (Table 4).

**Table 4:** Associations of BMI with antenatal depression, with interaction model between White British and South Asian women

Depression	White British		South Asian		Interaction AOR^a^ WB = reference
BMI categories^+^	Unadjusted OR (95% CI)	AOR (95% CI)^a^	Unadjusted OR (95% CI)	AOR (95% CI)^a^	
Recommended weight	*Reference group*	*Reference group*	*Reference group*	*Reference group*	0.79 (0.46–1.37)
Underweight	1.16 (0.76–1.78)	0.98 (0.63–1.54)	0.92 (0.70–1.22)	0.87 (0.65–1.16)	0.66 (0.32–1.37)
Overweight	0.97 (0.82–1.14)	1.00 (0.84–1.20)	1.17 (1.00–1.36)*	1.16 (0.99–1.36)	0.95 (0.55–1.65)
Obese	1.05 (0.89–1.25)	1.05 (0.87–1.26)	1.10 (0.94–1.28)	1.13 (0.96–1.33)	1.10 (0.71–1.71)
Class 1	0.97 (0.79–1.20)	1.00 (0.80–1.25)	1.11 (0.93–1.32)	1.15 (0.96–1.38)	0.93 (0.52–1.64)
Class 2	1.22 (0.93–1.59)	1.16 (0.87–1.56)	1.01 (0.79–1.29)	1.01 (0.78–1.30)	0.68 (0.36–1.28)
Class 3	1.07 (0.74–1.55)	1.01 (0.68–1.51)	1.20 (0.91–1.86)	1.32 (0.90–1.94)	–

*OR* odds ratio

*AOR* adjusted odds ratio

*CI* Confidence Interval

*WB* White British

^+^White British BMI categories: Underweight, < 18.5 kg/m^2^; recommended weight, 18.5–24.9 kg/m^2^; overweight, 25–29.9 kg/m^2^; obese, ≥30 kg/m^2^; obese class 1, 30.0–34.9 kg/m^2^; obese class 2, 35.0–39.9 kg/m^2^; obese class 3, ≥40.0 kg/m^2^ South Asian BMI categories: underweight, < 18.5 kg/m^2^; recommended weight, 18.5–22.9 kg/m^2^; overweight, 23–27.49 kg/m^2^; obese, ≥27.5 kg/m^2^; obese class 1, 27.5–32.49 kg/m^2^, obese class 2, 32.5–37.49 kg/m^2^, obese class 3, ≥37.5 kg/m^2^*Significant at 0.05 significance level (two-tailed)

-Insufficient data to run the model

^a^Adjusted for maternal age, maternal education, area of residence deprivation and maternal smoking

Table 5 shows the crude and adjusted ORs for screening positive for anxiety within each ethnic group for each BMI category and AORs for interaction analysis between White British and South Asian women. Univariate analysis showed that White British women with an overweight BMI had significantly higher odds of anxiety compared with White British women of recommended BMI (OR 1.21, 95% CI 1.03–1.42). This remained significant after adjusting for age, ethnicity, education level, smoking and deprivation (AOR 1.25, 95% CI 1.05–1.47). White British women with obesity also had higher odds of anxiety compared with recommended weight (OR 1.18, 95% CI 1.00–1.39). However, after adjustments this result was no longer significant (AOR 1.13, 95% CI 0.95–1.35). There was no significant association between anxiety and early pregnancy BMI among South Asian women. Interaction analysis found no significant association between ethnicity and antenatal anxiety within any BMI category (Table 5).

**Table 5:** Associations of BMI with antenatal anxiety, with interaction model between White British and South Asian women

Anxiety	White British		South Asian		Interaction AOR^a^ WB = reference
BMI categories^+^	Unadjusted OR (95% CI)	AOR (95% CI)^a^	Unadjusted OR (95% CI)	AOR (95% CI)^a^	
Recommended weight	*Reference group*	*Reference group*	*Reference group*	*Reference group*	1.18 (0.69–2.01)
Underweight	0.80 (0.52–1.22)	0.75 (0.48–1.17)	0.80 (0.52–1.22)	0.82 (0.61–1.09)	1.23 (0.60–2.52)
Overweight	1.21 (1.03–1.42)*	1.25 (1.05–1.47)**	1.09 (0.94–1.27)	1.06 (0.91–1.24)	1.05 (0.0–1.79)
Obese	1.18 (1.00–1.39)*	1.13 (0.95–1.35)	1.13 (0.97–1.31)	1.13 (0.96–1.33)	1.04 (0.85–1.29)
Class 1	1.13 (0.92–1.38)	1.09 (0.88–1.35)	1.13 (0.95–1.34)	1.15 (0.96–1.38)	1.28 (0.73–2.23)
Class 2	1.17 (0.90–1.52)	1.13 (0.85–1.49)	1.13 (0.89–1.44)	1.09 (0.84–1.40)	1.17 (0.63–2.15)
Class 3	1.43 (0.99–2.04)	1.29 (0.89–1.88)	1.11 (0.78–1.58)	1.10 (0.75–1.62)	–

*OR* odds ratio

*AOR* adjusted odds ratio

*CI* Confidence Interval

*WB* White British

^+^White British BMI categories: Underweight, < 18.5 kg/m^2^; recommended weight, 18.5–24.9 kg/m^2^; overweight, 25–29.9 kg/m^2^; obese, ≥30 kg/m^2^; obese class 1, 30.0–34.9 kg/m^2^; obese class 2, 35.0–39.9 kg/m^2^; obese class 3, ≥40.0 kg/m^2^ South Asian BMI categories: underweight, < 18.5 kg/m^2^; recommended weight, 18.5–22.9 kg/m^2^; overweight, 23–27.49 kg/m^2^; obese, ≥27.5 kg/m^2^; obese class 1, 27.5–32.49 kg/m^2^, obese class 2, 32.5–37.49 kg/m^2^, obese class 3, ≥37.5 kg/m^2^*Significant at 0.05 significance level (two-tailed)

**Significant at 0.01 significance level (two-tailed)

-Insufficient data to run the model

^a^Adjusted for maternal age, maternal education, area of residence deprivation and maternal smoking

Other independent variables were shown to be associated with antenatal depression and anxiety within White British and South Asian women (Table 6). For White British women, maternal age (AOR 0.98, 95% CI 0.97–0.99) and education (no education AOR 2.12, 95% CI 1.63–2.76) were significantly associated with depression, and smoking was significantly associated with both depression (AOR 1.32, 95% CI 1.12–1.56) and anxiety (AOR 1.34, 95% CI 1.14–1.57). For South Asian women, maternal age (AOR 1.02, 95% CI 1.01–1.04) and education (no education AOR 0.65, 95% CI 0.55–0.78) were significantly associated with anxiety, and smoking was significantly associated with both depression (AOR 2.08, 95% CI 1.49–2.91) and anxiety (AOR 2.87, 95% CI 2.02–4.07).

**Table 6:** Adjusted^a^ regression analysis for additional exposure variables association with depression and anxiety, stratified by ethnicity

	White British		South Asian	
Variables	Depression AOR (95% CI)	Anxiety AOR (95% CI)	Depression AOR (95% CI)	Anxiety AOR (95% CI)
Age	0.98 (0.97–0.99)*	1.00 (0.98–1.01)	1.00 (0.99–1.01)	1.02 (1.01–1.04)*
Education				
Higher than A-level	*Reference group*	*Reference group*	*Reference group*	*Reference group*
A-level	1.26 (0.97–1.62)	0.99 (0.79–1.25)	1.45 (1.18–1.77)**	1.31 (1.07–1.60)*
GCSE	1.54 (1.22–1.95)**	1.15 (0.93–1.42)	1.22 (1.04–1.43)*	0.93 (0.80–1.10)
None	2.12 (1.63–2.76)**	1.25 (0.98–1.59)	0.99 (0.83–1.77)	0.65 (0.55–0.78)**
IMD level				
Low deprivation	*Reference group*	*Reference group*	*Reference group*	*Reference group*
High deprivation	1.17 (1.00–1.37)	0.93 (0.80–1.08)	1.13 (0.97–1.31)	0.98 (0.85–1.14)
Smoking status				
No	*Reference group*	*Reference group*	*Reference group*	*Reference group*
Yes	1.32 (1.12–1.56)*	1.34 (1.14–1.57)**	2.08 (1.49–2.91)**	2.87 (2.02–4.07)**

*GCSE* The General Certificate of Secondary Education

*IMD* Index of Multiple deprivation

*AOR* adjusted odds ratio

*CI* Confidence Interval

*Significant at 0.05 significance level (two-tailed)

**Significant at 0.01 significance level (two-tailed)

^a^Adjusted for BMI, maternal age, maternal education, area of residence deprivation and maternal smoking

## Discussion

### Main findings

This study identified little evidence that early pregnancy BMI is a risk factor for antenatal depression or anxiety within South Asian and White British women in this population. There was an association between ethnicity and antenatal depression and anxiety with a significantly higher proportion of South Asian women with depression compared with White British women, but lower proportion with anxiety, independent of early pregnancy BMI.

Analysis of other socio-demographic variables identified smoking during pregnancy was a more important risk factor for both antenatal depression and anxiety in White British and South Asian women than early pregnancy BMI. Lack of education was a risk factor for antenatal depression in White British women but reduced the odds of antenatal anxiety in South Asian women. Older age was a risk factor for antenatal anxiety in South Asian women but reduced the odds of antenatal depression in White British women. However, effect sizes for maternal age were very small compared to smoking and education.

### Interpretations

Our results indicate there were no significant associations between antenatal depression and anxiety and early pregnancy BMI within this population. There is limited literature on the association between maternal BMI and depression and anxiety in the UK, with one study also showing no significant association in either mental health condition [26]. However, previous studies across international, mainly high-income, settings showed significantly increased odds of depression during pregnancy among women with overweight and obese BMIs, compared with women of recommended BMI [4–6]. The meta-anlaysis [4] also found significantly increased odds of anxiety in women with obesity, compared with women of recommended BMI, but no significant association for overweight.

The difference in statistical significance of the results between this study and previous literature could be due to the outcome measurement tool used. The GHQ was not used in any of the studies which mostly used either the Centre for Epidemiological Studies Depression Scale [5–7] for depression and The State-Trait Anxiety Inventory for anxiety [7]. This can introduce variation in the classification of depression and anxiety between this study and other literature.

In this study, depression rates are higher in South Asian women compared with White British, whereas, anxiety rates are lower. Gater et al. [27] found that depressive disorder was more common in Pakistani women compared with White women. Mckenzie et al. [28] found higher rates of suicide among older South Asian women, compared to White women. In relation to anxiety, there is a lack of published literature examining rates of anxiety within South Asian women in the UK. However, Weich et al. [3] found that rates of common mental disorders (anxiety and depression) were higher in Pakistani women compared to White women of similar age. The contradicting results for anxiety may be partly methodological, since Weich et al. [3] used the Revised Clinical Interview Schedule, whereas, this study used the GHQ which is not recommended for epidemiological assessment in South Asian women [29]. It may also be explained by the study settings; this study was set in Bradford which has a high density of South Asian’s. These large social networks have been shown to be beneficial for mental health due to feelings of shared identity and provision of support [30]. Therefore, in this study population, general anxiety symptoms may be managed by these large social networks among South Asian women.

Smoking during pregnancy has been associated with increased antenatal depression and anxiety in the literature [31, 32]. This may be due to the negative impact of nicotine on psychiatric symptoms [32] but also related to increased guilt for not being able to quit and health concerns for the baby [32]. Although this study found that less South Asian women smoke during pregnancy, the impact of smoking tends to be higher among South Asian women due to cultural and religious stigma attached to it [33]. This could play a role in higher depression rates found in South Asian women and should be effectively managed during antenatal care for South Asian women.

Lack of education has been shown to be a risk factor for antenatal depression in many studies [34–36]. However, in a study of South Asian women, it reduced antenatal anxiety and depression [37] which is also highlighted in this study. This could be explained by gender roles in South Asian culture in which women are expected to be more involved in taking care of the family than earning money or getting educated [38]. Therefore, South Asian women with less education tend to be more accepted by their in-laws as they fulfil their roles and responsibilities within the family. This may reduce the amount of stress and conflict within the family.

### Strengths and limitations

This study used data from a large prospective birth cohort, which minimises selection bias. However, following exclusions and categorisation of data, sample sizes were reduced. Although this study had a large sample size of 3514 White British women and 4310 South Asian women, the numbers of women with early pregnancy obesity were much smaller (903 and 1312 respectively). This study applied the Asian-specific BMI criteria as well as carrying out a secondary analysis using general population BMI criteria, which adds to the pregnancy evidence-base for South Asian women. We also used mother’s booking BMI which is measured in early pregnancy rather than self-reported pre-pregnancy weight and height. This approach reduces self-reporting bias since self-reported BMI tends to be under-estimated and provides a more accurate measure of BMI [39].

The South Asian ethnic group encompassed Pakistani, Indian and Bangladesh women which may introduce heterogeneity. Pakistani women have higher rates of obesity compared to Indian and Bangladeshi women [40]. Rates of depression are also different between these sub-groups, with Indians showing higher rates (61%) compared with Pakistani and Bangladeshi people (55%) [41]. Nevertheless, 88% of the included South Asian group were Pakistani, therefore, inclusion of women from India and Bangladesh may not have resulted in much bias.

The use of the GHQ in a South Asian sample comes with limitations as screening instruments may perform differently in different populations due to cultural and social differences [29]. This is especially important to consider when assessing differences between populations as was the case in this study. In a study examining the psychometric properties of the subscales of the GHQ in a multi-ethnic maternal sample from the BiB cohort [29], results showed that there was variation in the concepts measured by the GHQ between groups of different language and ethnic heritage. This may be due to the artefacts of translation and administration bias. The meaning of underlying concepts for some of the GHQ items differ according to language of administration [29]. Nevertheless, these issues of subjectivity and translations are found within most measurement tools, hence, more studies should be carried out in South Asian women using measurement tools such as the Edinburgh Postnatal Depression Scale which has validated Urdu [42] and Bengali [43] versions.

## Conclusions

This study suggests little evidence to support the known association between early pregnancy BMI and antenatal depression and anxiety in South Asian or White British women in this population. Smoking was found to be an important risk factor for antenatal depression and anxiety, particularly among South Asian women who have higher rates of antenatal depression. This has implications on the antenatal care South Asian women receive which should acknowledge cultural stigma associated with smoking. Further research should focus on South Asian women using validated depression and anxiety measurement tools for this population and Asian-specific BMI criteria.

## Supplementary information

**Additional file 1.** General Health Questionnaire: List of all 28 GHQ questions and the subscales.

**Additional file 2: Table S1.** Associations of BMI with antenatal depression in South Asian women using the general population BMI criteria.

## Data Availability

The data that support the findings of this study are available from Born in Bradford research committee, but restrictions apply to the availability of these data, which were used under license for the current study, and so are not publicly available. Data are however available from the authors upon reasonable request and with permission of Born in Bradford.

## Abbreviations

AOR: Adjusted Odds Ratio
BiB: Born in Bradford
BMI: Body Mass Index
CI: Confidence Interval
GHQ: General Health Questionnaire
IMD: Index of Multiple Deprivation
WHO: World Health Organisation
