# Supplementary material for: Antenatal depression and anxiety and early pregnancy BMI among White British and South Asian women: retrospective analysis of data from the Born in Bradford cohort
Source: BMC Pregnancy Childbirth. 2020 Sep 1;20:502. doi: 10.1186/s12884-020-03097-2 (PMC7466782; doi:10.1186/s12884-020-03097-2)
Supplement: Supplementary file 1 — Additional file 1. General Health Questionnaire: List of all 28 GHQ questions and the subscales. [file 12884_2020_3097_MOESM1_ESM.docx]

**Additional File 1**

**General Health Questionnaire (GHQ)**

**Subscale A items 1-7 (somatic symptoms)**

1. Been feeling perfectly well & in good health
2. Been feeling in need of a good tonic
3. Been feeling run down and out of sorts
4. Felt that you were ill
5. Been getting pains in your head
6. Been getting a feeling of tightness or pressure in your head
7. Been having hot or cold spells

**Subscale B items 8-14 (anxiety/insomnia)**

1. Lost much sleep over worry
2. Had difficulty staying asleep once you are off
3. Feeling constantly under strain
4. Been getting edgy and bad-tempered
5. Been getting scared or panicky for no good reason
6. Found everything getting on top of you
7. Been feeling nervous and strung-up all the time

**Subscale C items 15-21 (social dysfunction)**

1. Been managing to keep yourself busy and occupied
2. Been taking longer over the things you do
3. Felt on the whole you were doing things well
4. Been satisfied with way you’ve carried out your tasks
5. Felt that you are playing a useful part in things
6. Felt capable of making decisions about things
7. Been able to enjoy your normal day-to-day activities

**Subscale D items 22-28 (severe depression)**

1. Been thinking of yourself as a worthless person
2. Felt life is entirely hopeless
3. Felt that life isn’t worth living
4. Thought of the possibility that you might make away with yourself
5. Found at times couldn’t do anything because nerves too bad
6. Found yourself wishing you were dead and away from it all
7. Found the idea of taking your own life kept coming into your mind

**Each item coded on a Likert scale**

1. Not at all
2. No more than usual
3. Rather more than usual
4. Much more than usual
