# Supplementary material for: Antenatal depression and anxiety and early pregnancy BMI among White British and South Asian women: retrospective analysis of data from the Born in Bradford cohort
Source: BMC Pregnancy Childbirth. 2020 Sep 1;20:502. doi: 10.1186/s12884-020-03097-2 (PMC7466782; doi:10.1186/s12884-020-03097-2)
Supplement: Supplementary file 2 — Additional file 2: Table S1. Associations of BMI with antenatal depression in South Asian women using the general population BMI criteria. [file 12884_2020_3097_MOESM2_ESM.docx]

**Table S1** Associations of BMI with antenatal depression in South Asian women using the general population BMI criteria^+^

|  | **Depression** | | **Anxiety** | |
| --- | --- | --- | --- | --- |
| **BMI categories** | Unadjusted OR (95% CI) | **AOR (95% CI)^a^** | Unadjusted OR (95% CI) | **AOR (95% CI)^a^** |
| **Recommended weight** | *Reference group* | ***Reference group*** | *Reference group* | ***Reference group*** |
| **Underweight** | 0.87 (0.66-1.14) | **0.83 (0.62-1.09)** | 0.76 (0.58-0.99)* | **0.79 (0.60-1.05)** |
| **Overweight** | 1.07 (0.93-1.24) | **1.08 (0.94-1.26)** | 1.04 (0.90-1.19) | **1.04 (0.90-1.21)** |
| **Obese** | 1.02 (0.87-1.21) | **1.06 (0.89-1.26)** | 1.05 (0.89-1.24) | **1.07 (0.89-1.27)** |
| **Obese class 1** | 1.00 (0.82-1.21) | **1.05 (0.86-1.29)** | 1.10 (0.91-1.34) | **1.11 (0.91-1.36)** |
| **Obese class 2** | 0.98 (0.73-1.31) | **0.98 (0.72-1.33)** | 0.95 (0.71-1.27) | **0.97 (0.71-1.33)** |
| **Obese class 3** | 1.43 (0.87-2.34) | **1.48 (0.87-2.53)** | 0.99 (0.61-1.62) | **0.97 (0.57-1.66)** |

OR=odds ratio

AOR=adjusted odds ratio

^+^ Underweight, <18.5 kg/m^2^; recommended weight, 18.5-24.9 kg/m^2^; overweight, 25-29.9 kg/m^2^; obese, ≥30 kg/m^2^; obese class 1, 30.0-34.9 kg/m^2^; obese class 2, 35.0-39.9 kg/m^2^; obese class 3, ≥40.0 kg/m^2^

^a^Adjusted for maternal age, maternal education, area of residence deprivation and maternal smoking

*Significant at 0.05 significance level (two-tailed)
